# Supplementary material for: SMRT-Cappable-seq reveals complex operon variants in bacteria
Source: Nat Commun. 2018 Sep 10;9:3676. doi: 10.1038/s41467-018-05997-6 (PMC6131387; doi:10.1038/s41467-018-05997-6)
Supplement: Supplementary file 1 — Supplementary Information [file 41467_2018_5997_MOESM1_ESM.pdf]

SMRT-Cappable-seq reveals complex operon variants in bacteria

Bo et al.

Supplementary Information

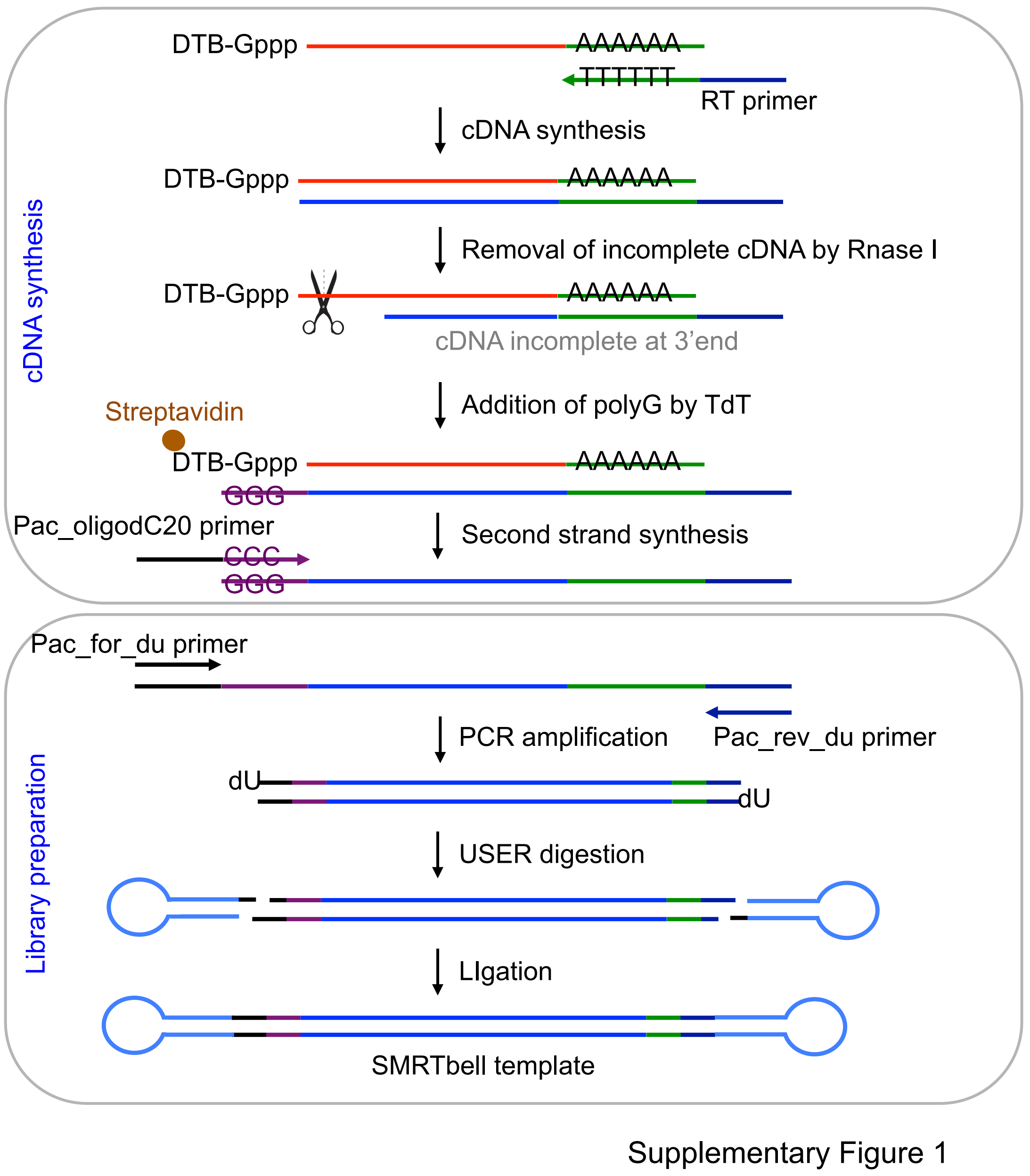

Supplementary Figure 1: Schema of the SMRT-Cappable-seq library preparation.

First strand cDNA is synthesized using RT primer containing dT sequence. The RT step is followed by Rnase I treatment to remove incomplete cDNA fragments. Terminal transferase is then used to add a polyG tail at the 3' end of the cDNA. Second strand cDNA is made using a polyC anchored primer (Pac_oligodC20 primer) and further amplified by PCR. The dUracil in the primer is removed using USER to create sticky ends, and PacBio adapters are ligated to the amplified fragments to generate SMRTbell templates. SMRT sequencing of the resulting SMRTbell templates provides the genome-wide definition of full-length transcripts at base resolution.

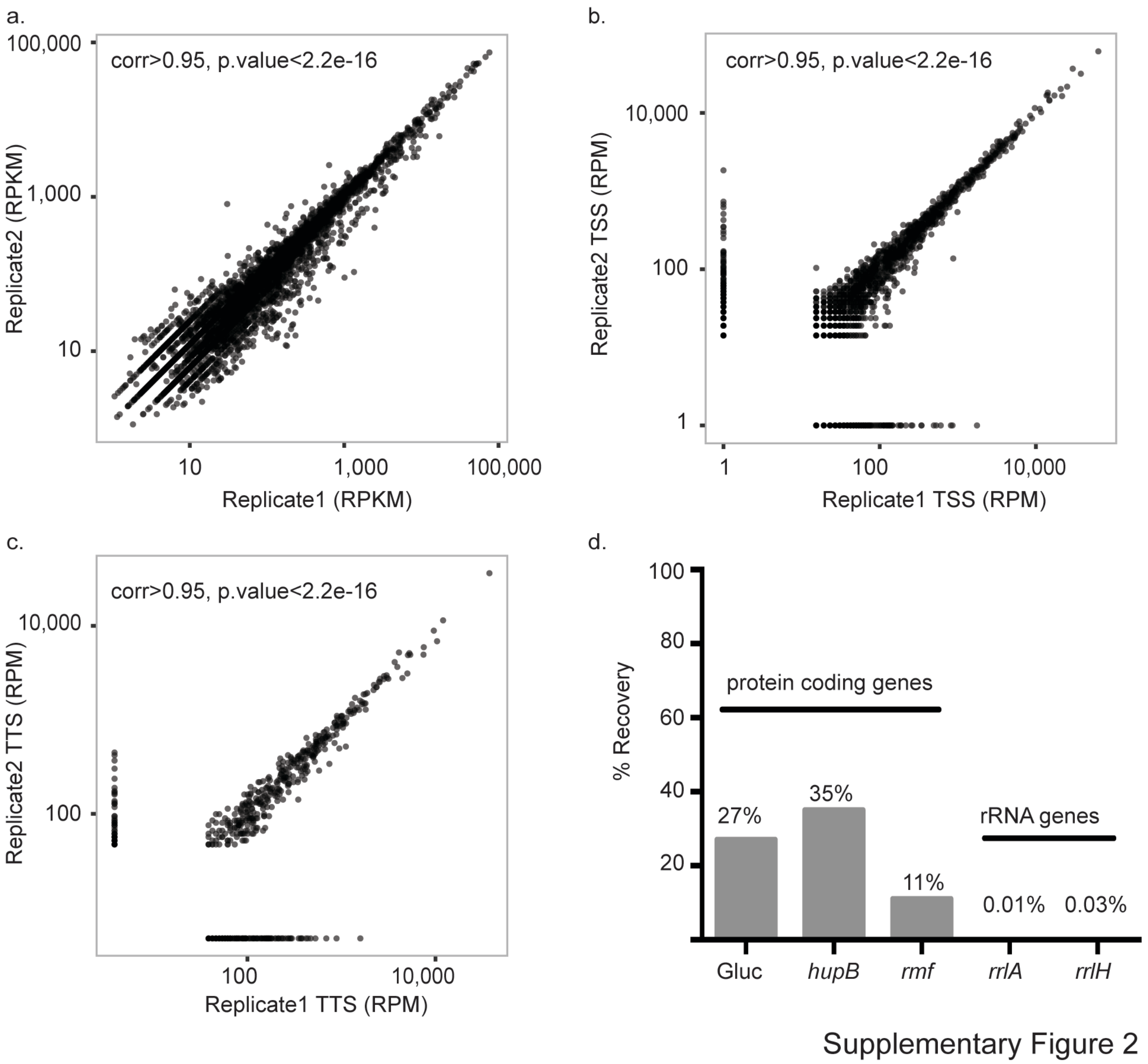

Supplementary Figure 2: SMRT Cappable-seq quality controls.

Correlation between two technical replicates in terms of gene expression (**A**), TSS usage (**B**) and TTS usage (**C**). Gene expression is measured in Read counts Per Kilobase of transcript, per Million mapped reads (RPKM); TSS and TTS usage are measured in Read counts Per Million mapped reads (RPM). **D.** Recovery of primary and processed transcripts after SMRT-Cappable-seq: The mRNA levels of protein coding genes (Gluc, *hupB* and *rmf*) and rRNA genes (*rrlA* and *rrlH*) were measured by qPCR using the cDNA obtained from *E. coli* grown in M9 medium. The recovery (in %, Y axis) was calculated as the amount of mRNA in the enriched group (after streptavidin) divided by the amount of mRNA in the control group (no streptavidin enrichment). Gluc is an *in-vitro* transcribed mRNA spiked in the *E. coli* total RNA as positive 5' triphosphorylated control. *hupB* and *rmf* are endogenous protein coding genes representative of primary transcripts. *rrlA* and *rrlH* are rRNA genes representative of processed transcripts. The average recovery rate for primary and processed transcript is 24% and 0.02%, respectively.

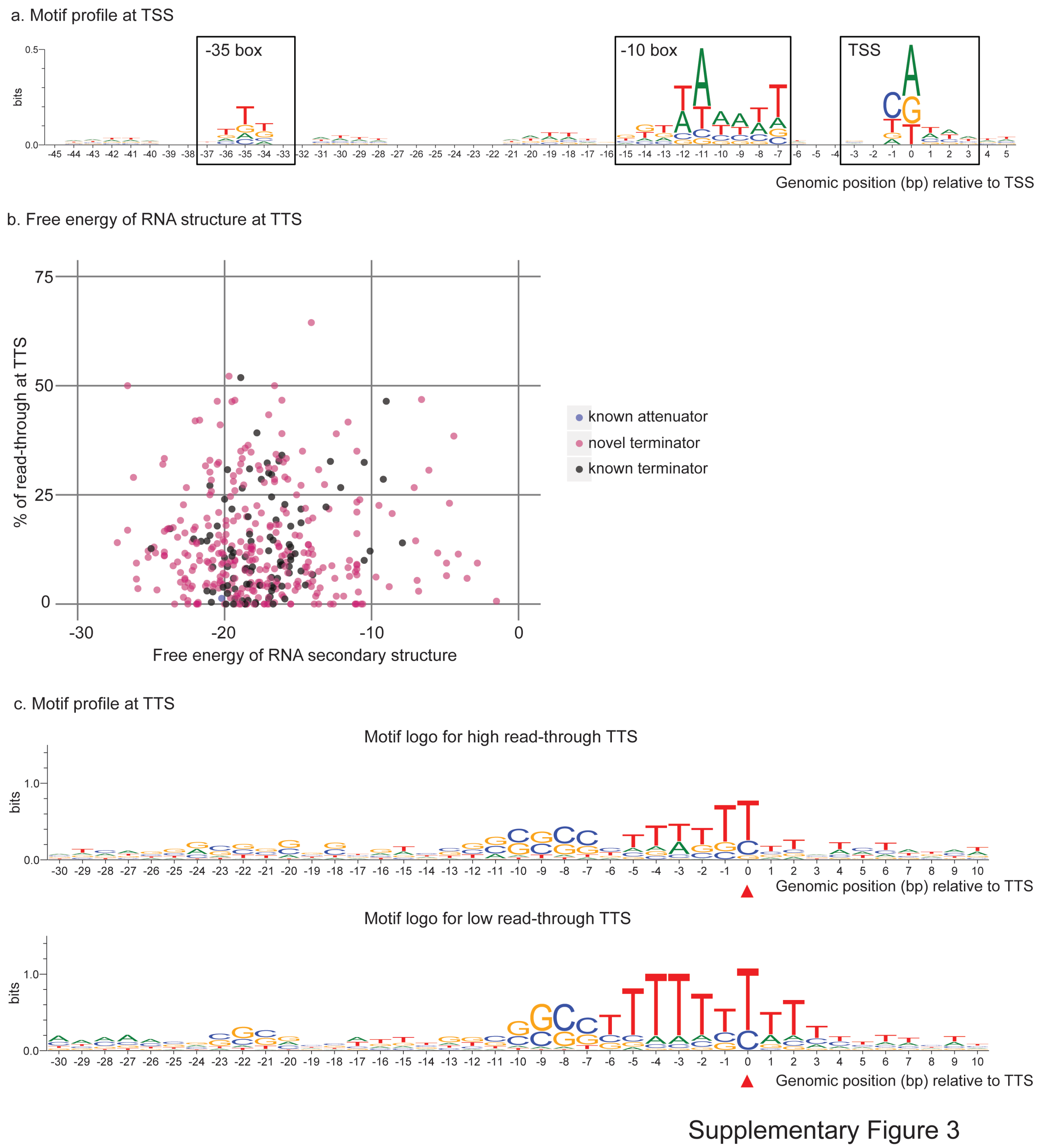

Supplementary Figure 3: Motif and RNA structure analysis for TSS and TTS in M9 medium.

A. Motif logos for SMRT-Cappable-seq defined TSSs. X-axis: Position 0 corresponds to TSS, negative and positive values correspond to positions upstream and downstream of TSS respectively. Y-axis: information content expressed in bits. **B.** No significant correlation

(Pearson corr=0.022, p=0.6487) between the degree of read-through of the TTS and the free energy of the RNA structure (X-axis) of the TTS can be observed. **C.** Motif logos for TTSs (top) that have high (more than 25%) read-through and for TTSs (bottom) that have low (less than 25%) read-through. Red arrow indicates the termination site. X-axis: Predicted TTS is located at position 0, negative and positive values correspond to positions upstream and downstream of TTS respectively. Y-axis: information content expressed in bits.

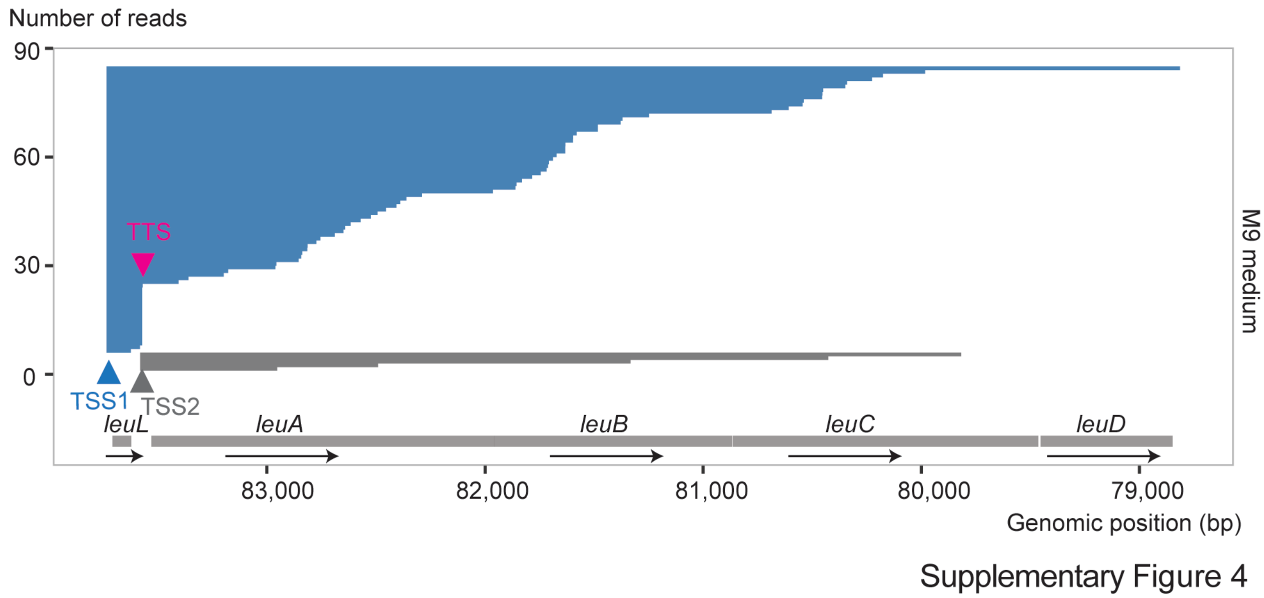

Supplementary Figure 4: Structure of the Leu operon.

Individual mapped reads ordered by TSS location and read size in the Leu operon locus. The red arrow denotes the termination site controlled by a riboswitch. Reads in blue represent transcripts originated from the TSS1 upstream of the leader peptide *leuL* (pos=83735). Reads in grey represent transcripts originated from the TSS2 downstream of the leader peptide (pos=83581).

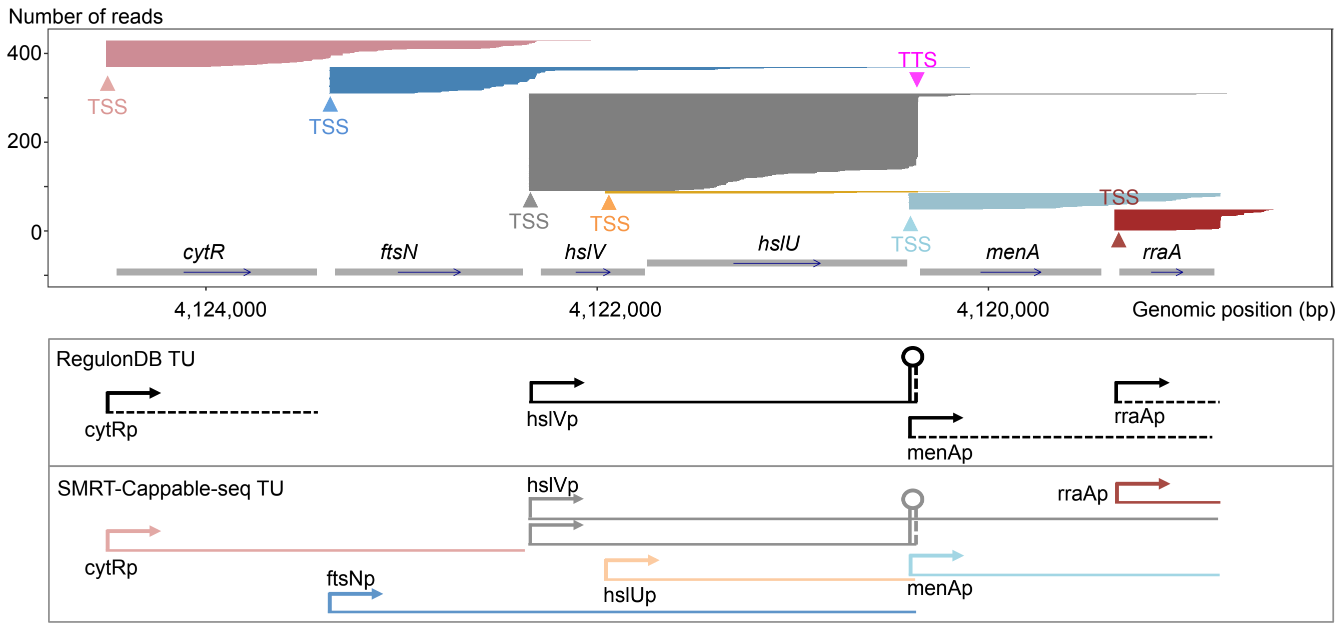

Supplementary Figure 5

Supplementary Figure 5: SMRT-Cappable-seq read profile in Rich condition.

Schematic representation of previously annotated operons from RegulonDB database (dash line indicates weak evidence, and solid line indicates strong evidence) and operons defined by SMRT-Cappable-seq. There are four annotated operons covering the coding genes in the shown genome region, three of which are also defined by SMRT-Cappable-seq (*hslV-hslU*, *menA-rra*, *rra*). SMRT-Cappable-seq additionally identifies four extended operons. Two of them (*ftsN-hslV-hslU* and *hslU*) have previously unidentified promoters and 5' genes. The other *cytR-ftsN* has the same promoter as the previous *cytR* unit, but includes an additional gene at the 3'end. Another extended operon is composed of the *hslV-hslU-menA-rraA* genes due to the read-through at the *hslV-hslU* terminator. Red arrow indicates the previously known TTS for the operon.

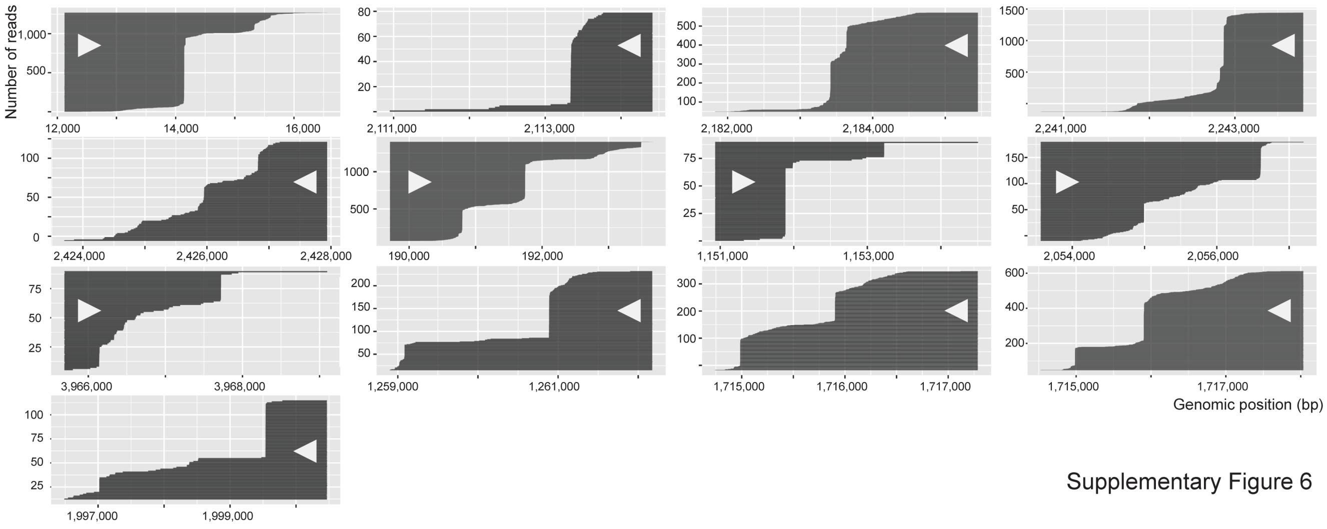

Supplementary Figure 6

Supplementary Figure 6: Sequential read-through across TTS leads to staircase patterns.

Examples of staircase patterns generated from transcripts sharing the same TSS as a result of sequential read-through at termination sites. Data shown is from M9 growth condition. Arrows denote the direction of transcription.

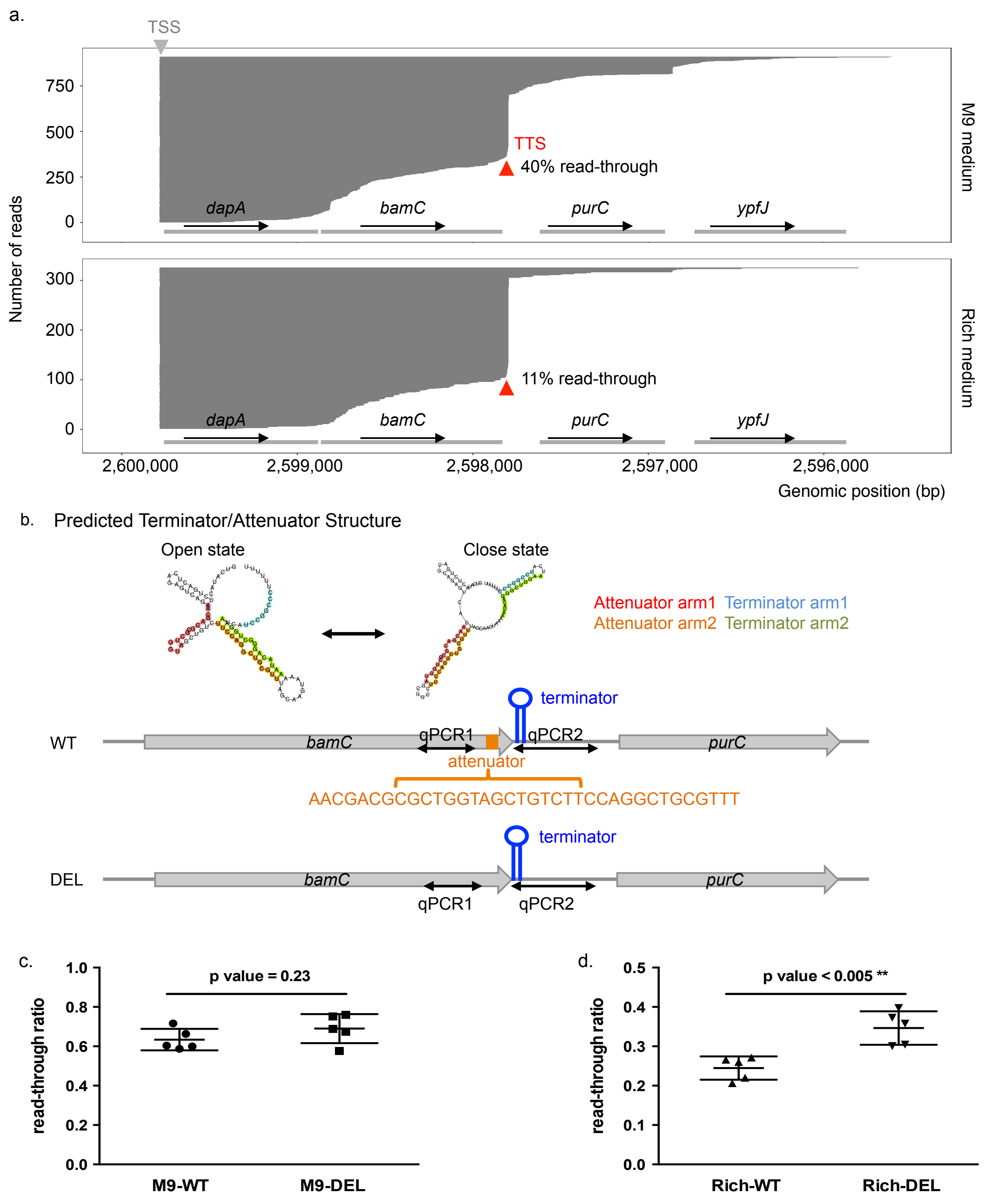

Supplementary Figure 7

Supplementary Figure 7: The attenuator structure controls the condition dependent read-through across the *dapA-bamC* termination site.

A. An example of the *dapA-ypfJ* operon containing the previously known *dapA-bamC* TTS (red arrow), where the read-through is condition dependent. **B**. Schema of this TTS location. The attenuator structure (orange) that locates 10 bp upstream of the termination site (blue) is predicted and deleted to examine the role of this regulatory region in the control of transcription termination. **C. D.** The levels of read-through across the TTS of wild-type (WT) and deletion strain (DEL) were measured for bacteria grown in both M9 and Rich medium by qPCR. The qPCR1 primers amplify an upstream region of the predicted attenuator site. For qPCR product2, the forward primer binds to the 5'end of the known *dapA-bamC* TTS while the reverse primer binds to the downstream region of the TTS. Therefore, read-through ratio was calculated as the amount of qPCR2 product divided by the amount of qPCR1 product. Data shown are the means ± SD from five independent repeats. Unpaired t-test was used to determine significance. The read-through in DEL is significantly higher than in WT in Rich condition.

Supplementary Table 1: Sequences of primers used in this study.

Primer Name	Sequence
Primers used for Library Preparation	
RT primter	GTGACTGGAGTTCAGACGTGTGCTCTTCCGATCTNNNNNNGCG CTTTTTTTTTTTTTTTTTTTVN
Pac_oligodC20	ACACTCTGTCGCTACGTAGATAGCGTTGAGTGCCCCCCCCCCCC CCCCCCCC
Pac_for_dU	G/ideoxyU/ACACTCTGTCGCTACGTAGATAGCGTTGAGTG (AdapterL sequence)
Pac_rev_dU	G/ideoxyU/GTGACTGGAGTTCAGACGTGTGCTCTTCCGATCT
PacBio adapter	/5Phos/ATCTCTCTCTTTTCCTCCTCCTCCGTTGTTGTTGTTGAGAG AGATTGT
Primers used for qPCR	
Gluc_forward	CGACATTCCTGAGATTCCTGG
Gluc_reverse	TTGAGCAGGTCAGAACACTG
rrlH_forward	AGTGGAAGCGTCTGGAAGG
rrlH_reverse	GCCCTAGTCATCGAGCTCAC
rrlA_forward	ACAAAAACGAGATCGCCTGGA
rrlA_reverse	CGAAGTTACGGCACCATTTT
hupB_forward	TTTGCCGTTAAAGAGCGTGCT
hupB_reverse	TACCGCGTCTTTCAGTGCTT
rmf_forward	ACAAAAACGAGATCGCCTGGA
rmf_reverse	CAGCGTCTGATAGGGACACA
qPCR1_forward	GCATGAAAGTGACCGACAGC
qPCR1_reverse	GTAGGCTGCTGCGGTTATCT
qPCR2_forward	AATACAGGGCTGGAATCATCC
qPCR2_reverse	CCGACAAACAAATTTCGTGCG

Supplementary Note 1: Comparison between Illumina and PacBio data

In order to investigate whether PacBio data can quantitatively estimate the expression level of transcripts, we compared the gene expression levels measured using SMRT-Cappable-seq and standard RNA-seq from Illumina. For this, we used a previously published RNA-seq experiment done on *E. coli* K12 strain MG1655 grown in similar conditions as our experiments (M9 medium) [1]. We estimated gene expression level (in RPKM) for both the Illumina dataset and the SMRT-Cappable-seq dataset based on the number of reads overlapping with annotated genes (**Methods**). At the gene level, the correlation between Illumina and PacBio dataset is estimated to be 0.798 ($p<2.2e-16$) using Spearman's rank correlation.

Supplementary Note 2: Comparison between biological replicates

In order to measure the reproducibility, we prepared two SMRT-Cappable-seq libraries using 5 μg of *E. coli* RNA from M9 medium as biological replicates. These two libraries were sequenced using PacBio Sequel platform as mentioned in Methods, and we generated around 0.2 million reads for each replicate. For both replicates, we estimated gene expression level and counted the number of reads at TSS and TTS. The correlation between two replicates is greater than 0.95 estimated using Pearson correlation (Supplementary Fig. 2a, 2b and 2c).

Supplementary Note 3: Comparison of SMRT-Cappable-seq reads with processed/primary rRNA

For rRNA analysis, SMRT-Cappable-seq reads were mapped to *E. coli* U00096.2 genome and classified based on the overlap with its annotated genomic features. Overlap calculations were performed using bedtools intersect version v2.24.0 (-s parameter). Reads mapped to *E. coli* genome were classified into 3 categories (Fig. 1d): (1) starting at the known primary rRNA TSS sites [2] (primary rRNA); (2) overlapping with the rRNA genes but do not start at the primary TSS sites (processed rRNA); (3) overlapping with other coding genes (protein coding gene). Here we distinguished processed and primary rRNA because primary rRNA transcripts are expected to be enriched in the SMRT-Cappable-seq library compared to control, while the processed rRNA should be depleted.

Mappable reads can be classified into one of these categories with the following result:

For *E. coli* grown under M9 condition, in SMRT-Cappable-seq library, from 279298 total mappable reads, 13621 reads start at the TSSs of primary rRNA transcripts (4.87%), 12170 reads are processed rRNA (4.35%) and 253507 are assigned to other protein coding genes (90.75%) .

For *E. coli* grown under Rich condition, in SMRT-Cappable-seq library, from 249772 total mappable reads, 21290 reads start at the TSSs of primary rRNA transcripts (8.52%), 24591 (9.84%) reads are processed rRNA and 203891 are assigned to other protein coding genes (81.64%).

Supplementary Note 4: Comparison of SMRT-Cappable-seq with reported 5' and 3' processing sites

We compared the SMRT-Cappable-seq transcripts with the reported 5' processing sites of rRNA or tRNA genes and the 3' processing ends of Rnase III cleavage sites. Compared with the 2054 previously reported Rnase III cleavage sites [3], for M9 condition, only 7 out of 347 SMRT-Cappable-seq TTSs were within +/- 10 bp of a Rnase III cleavage site. These sites include the 3' processed ends of 4 rRNA genes: *rrfG* (genome position 2726273), *rrlD* (3423873), *rrsH* (223653), and *rrlE* (4212954). For Rich condition, there were 376 unique TTSs and 11 were within +/- 10 bp of a Rnase III cleavage site.

Supplementary Note 5: Comparison of SMRT-Cappable-seq reads with Cappable-seq TSS

TSSs from both SMRT-Cappable-seq and control libraries were compared to the published TSS data obtained under the same growth condition (M9, log phase) [2]. Because SMRT-Cappable-seq library preparation procedure involves the non-template addition of nucleotides, the exact position of the read start can be off by one or several nucleotides. For this, we extended the Cappable-seq clustered TSS by 10 bp (+5bp to -5bp) and calculated the percentage of SMRT-Cappable-seq and control reads that start within the extended Cappable-seq TSS clusters.

We found that there were 258849 (93%) SMRT-Cappable-seq reads starting within the 10 bp region of the Cappable-seq clustered TSSs, while this number was only 30959 (15%) for control.

Supplementary Note 6: Comparison of SMRT-Cappable-seq TTS with previously annotated TTS

To compare TTSs with the previously experimentally identified termination sites and attenuator sites from Ecocyc [4], we measured the distance of SMRT-Cappable-seq TTS to its nearest known termination site. Because SMRT-Cappable-seq library preparation procedure involves the non-template addition of A at the 3'end of the RNA, the exact position of the read start can be off by one or several nucleotides in the case of a TTS finishing at A. Since the length of annotated terminators is around 10 to 70 bp, the TTSs that have distance shorter than 50 bp to a known TTS site were reported as previously known terminators, and the others were reported as novel sites.

Supplementary Note 7: Calculation of read-through at termination sites

Because TTS read-through can be TSS dependent, we use TSS-TTS pair to calculate the read-through. For M9 condition, we identified a total of 408 TSS-TTS pairs corresponding to 347 unique TTSs. Amongst the 347 TTSs, 75 were previously annotated as Rho-independent TTS and one (*artJ* terminator) was previously annotated as Rho-dependent (Supplementary Data 1). For Rich condition, we identify a total of 455 TSS-TTS pairs corresponding to 376 unique TTSs.

Amongst those TTSs, 76 TTSs were previously annotated as Rho-independent TTS (Supplementary Data 1).

To calculate the read-through for each TSS-TTS pair, we calculated (A) the number of reads starting and ending at the TSS and TTS (Supplementary Data 1 column 6); and (B) the number of reads starting at the same TSS but ending at any position within the region 50 bp downstream of the TTS (Supplementary Data 1 column 8); (C) To avoid false positives, only reads that are extended by least 50 bp downstream of the TTS are defined as read-through (Supplementary Data 1 column 7). Accordingly, we define the percentage of read-through across a TTS as the ratio of (C) divided by the sum of (A), (B) and (C). For M9 condition, the read-through is above 5% for 305 out of 408 TSS-TTS pairs, and 40% (151 from 408) of TSS-TTS pairs have read-through transcripts that include additional gene(s).

Supplementary Note 8: SMRT-Cappable-seq operons

We combined both M9 and Rich datasets to define the operons (Methods), and a total of 2347 operons were defined. They were compared with the RegulonDB operons and thereby classified into 4 categories (Supplementary Data 2): (1) having the same 5'end and 3'end gene (same, in total 1253); (2) having the same 5'end gene but novel 3'end gene that was defined by the TTS (BinomialEnd, in total 55); (3) having the same 5'end gene but a 3'end gene within a known operon, which means the defined operon was shorter at 3'end (ShorterEnd, in total 254); (4) having novel 5'end gene or 3'end gene outside a known operon (Novel, in total 785). Importantly, the operons in category (2) and (4) are not annotated before.

Supplementary Reference

1. Vital M, Chai B, Ostman B, Cole J, Konstantinidis KT, Tiedje JM: Gene expression analysis of E. coli strains provides insights into the role of gene regulation in diversification. *ISME J* 2015, 9:1130-1140.
2. Ettwiller L, Buswell J, Yigit E, Schildkraut I: A novel enrichment strategy reveals unprecedented number of novel transcription start sites at single base resolution in a model prokaryote and the gut microbiome. *BMC Genomics* 2016, 17:199.
3. Gordon GC, Cameron JC, Pfleger BF: RNA Sequencing Identifies New RNase III Cleavage Sites in Escherichia coli and Reveals Increased Regulation of mRNA. *MBio* 2017, 8.
4. Keseler IM, Mackie A, Peralta-Gil M, Santos-Zavaleta A, Gama-Castro S, Bonavides-Martinez C, Fulcher C, Huerta AM, Kothari A, Krummenacker M, et al: EcoCyc: fusing model organism databases with systems biology. *Nucleic Acids Res* 2013, 41:D605-612.
