# Supplementary material for: SMRT-Cappable-seq reveals complex operon variants in bacteria
Source: Nat Commun. 2018 Sep 10;9:3676. doi: 10.1038/s41467-018-05997-6 (PMC6131387; doi:10.1038/s41467-018-05997-6)
Supplement: Supplementary file 3 — Description of Additional Supplementary Files [file 41467_2018_5997_MOESM3_ESM.pdf]

Description of Additional Supplementary Files

File Name: Supplementary Data 1
Description: TTSs defined by SMRT-Cappable-seq under M9 and Rich condition.
Column 1: Genome version.
Column 2 : TSS for forward strand OR TTS for reverse strand.
Column 3: TSS for reverse strand OR TTS for forward strand.
Column 4: Strand.
Column 5: The number of reads starting at the TSS position.
Column 6: The number of reads starting at the TSS and ending at the TTS.
Column 7: The number of reads starting at the TSS but passing through the TTS (which means ending at least 50 bp downstream of the TTS).
Column 8: The number of reads starting at the TSS and ending within the region 50 bp downstream of the TTS.
Column 9: Median size of the 3' extended region (bp).
Column 10: TTS types (novel TTS, known terminator or known attenuator).
Column 11: Name of the nearest known terminator.
Column 12: The position of the known terminator (center).
Column 13 : List of genes fully covered by the transcript (separated by |).

File Name: Supplementary Data 2
Description: SMRT-Cappable-seq operons.
Column 1: List of genes (separated by |) in the SMRT-Cappable-seq defined operon.
Column 2: Transcription start site of the SMRT-Cappable-seq operon.
Column 3: Operon strand.
Column 4: Comparison with RegulonDB. See Supplementary Notes for details.
Column 5: List of previously annotated RegulonDB operons that overlap with the SMRT-Cappable-seq operon (Separated by semi-colon) .

File Name: Supplementary Data 3
Description: RegulonDB operons extended by SMRT-Cappable-seq.
Column 1: List of genes in RegulonDB annotated operons (separated by |).
Column 2: List of genes contained by SMRT-Cappable-seq transcripts (separated by |).

File Name: Supplementary Data 4
Description: Transcriptional Context (TC).
Column 1: Gene ID.
Column 2: The number of TC that the gene is found in under M9 condition.
Column 3: The number of TC that the gene is found in under Rich condition.
